# High-Speed and Unified ECC Processor for Generic Weierstrass Curves over GF(*p*) on FPGA

**DOI:** 10.3390/s21041451

**Published:** 2021-02-19

**Authors:** Asep Muhamad Awaludin, Harashta Tatimma Larasati, Howon Kim

**Affiliations:** 1 School of Computer Science and Engineering, Pusan National University, Busan 609735, Korea; asep.muhamad11@pusan.ac.kr (A.M.A.); harashta@pusan.ac.kr (H.T.L.); 2 School of Electrical Engineering and Informatics, Institut Teknologi Bandung, Bandung 40116, Indonesia

**Keywords:** elliptic curves cryptography (ECC), high speed implementation, unified, Montgomery multiplication, field-programmable gate array (FPGA)

## Abstract

In this paper, we present a high-speed, unified elliptic curve cryptography (ECC) processor for arbitrary Weierstrass curves over GF(p), which to the best of our knowledge, outperforms other similar works in terms of execution time. Our approach employs the combination of the schoolbook long and Karatsuba multiplication algorithm for the elliptic curve point multiplication (ECPM) to achieve better parallelization while retaining low complexity. In the hardware implementation, the substantial gain in speed is also contributed by our *n*-bit pipelined Montgomery Modular Multiplier (pMMM), which is constructed from our *n*-bit pipelined multiplier-accumulators that utilizes digital signal processor (DSP) primitives as digit multipliers. Additionally, we also introduce our unified, pipelined modular adder/subtractor (pMAS) for the underlying field arithmetic, and leverage a more efficient yet compact scheduling of the Montgomery ladder algorithm. The implementation for 256-bit modulus size on the 7-series FPGA: Virtex-7, Kintex-7, and XC7Z020 yields 0.139, 0.138, and 0.206 ms of execution time, respectively. Furthermore, since our pMMM module is generic for any curve in Weierstrass form, we support multi-curve parameters, resulting in a unified ECC architecture. Lastly, our method also works in constant time, making it suitable for applications requiring high speed and SCA-resistant characteristics.

## 1. Introduction

The advances in technology have resulted in the emergence of various applications, such as 5G and blockchain-based services [1,2]. In most cases, acquiring high speed and low latency without compromising security aspects has become of great importance. Hence, elliptic curve-based cryptography (ECC) has become prominent in modern cryptography compared to the Rivest–Shamir–Adleman (RSA) due to its smaller key size for an equivalent security level [3]. Several protocols based on ECC are the Elliptic Curve Diffie–Hellman (ECDH) for the key agreement, as well as Elliptic Curve Digital Signature Algorithm (ECDSA), which is used extensively in the current digital signature schemes.

Among the existing ECC protocols, the use of the Weierstrass curve remains prevalent. In fact, this curve has still been widely adopted in the current implementations, ranging from blockchain-based applications to 5G services. For instance, Bitcoin, Ethereum, and Zcash employ the secp256k1 curve for their signature verification [4] while public-key schemes based on SM2 remain the standard for use in electronic authentication systems, key management, and e-commercial applications within China [5,6]. Additionally, Transport Layer Security (TLS) as the favored protocol for securing 5G communications, employs ECDH in its handshake process [7].

Since improving the performance of ECC is essential, several methods have been proposed to speed up the computation of the protocol. One of the techniques is by utilizing special primes (also known as generalized Mersenne primes), as recommended by the National Institute of Standards and Technology (NIST) [8], which greatly simplifies the modular reduction operation. Another approach is by employing efficiently-computable endomorphisms [9] to accelerate elliptic curve point multiplication (ECPM) in the curves with special properties (e.g., secp256k1), such as by using the Gallant–Lambert–Vanstone (GLV) method [10].

However, these schemes are specific to each modulus and curve’s domain parameters. Even though a very fast computation can be achieved, it comes with a huge trade-off in flexibility for the hardware implementation. This drawback is undesirable because in real-life use, we may need to employ more than one curve to facilitate different purposes. For instance, a web server may require multiple curves to comply with different security requirements among various platforms. Furthermore, recent applications of ECC have explored a nonstandard prime field that does not make use of a specific prime structure [11], such as the post-quantum supersingular isogeny-based key exchange (SIKE) algorithm [12] and bilinear pairing [13].

To maintain hardware implementation flexibility, several methods in literature have proposed to accelerate ECC computation for generic curves rather than a special curve, including [11,14,15,16,17,18,19,20]. In 2013, Ma et al. [16] proposed a generic ECC processor, which leverages the combination of a quotient-pipelining Montgomery multiplication with a parallel array design. Their technique, implemented on Virtex-5, yields a speed of 0.380 ms, which can be considered the fastest among other proposals. Other works on a more recent platform (e.g., Xilinx 7-series) can be found in [11,14,15]. Specifically, Asif et al. [14] utilized a residue number system (RNS) based ECC processor whereas Bajard et al. [15] leveraged a Montgomery Cox-Rower architecture, which gives a relatively lower speed of 0.730 and 0.612, respectively.

Recently, Roy et al. [11] proposed a fast implementation of ECC multiplication that works for arbitrary Montgomery curves using DSP cores on modern FPGA. Their proposed modular multiplier gives a competitive result: around 0.343 ms for the low area, and 0.39 ms for the single-core implementation, making their paper the state-of-the-art ECC processor for generic Montgomery curves. Additionally, the authors also provide the extension to the generic Weierstrass curve, which yields a slightly lower speed of 0.459 ms. However, their technique has dependent iterative variables, making further optimizations (e.g., pipelining method) infeasible. Using their approach, multiple cores will need to be used when dealing with simultaneous execution of several multiplications.

Since the performance of an ECC processor mostly depends on the underlying modular multiplication operation, especially when the point operation is optimized using a Jacobian coordinate to avoid modular inversion during ladder operation, an efficient multiplication technique will significantly increase the speed of the processor. The support for pipelining and parallelization, for example, may give a considerable speed increase in the hardware implementation. To date, one of the most favorable methods for multiplication is the Karatsuba–Ofman multiplication [21] since it offers a relatively low complexity. However, it would be very difficult to employ parallelization due to its recursive approach when dealing with higher bit length. On the other hand, the naïve way to perform multiplication is the schoolbook long multiplication, which scales quadratically in terms of complexity. Nevertheless, all digit multiplications can be executed in parallel, which can be efficiently implemented in the high-performance hardware by adopting a divide-and-conquer method.

In our study, we find that combining these two methods for our multiplication enables us to perform better parallelization, which in turn brings a substantial gain in speed for the FPGA implementation. Furthermore, we design our ECC architecture to support pipelining for achieving an even higher speed. In particular, the speed-up is mainly contributed by our *n*-bit pipelined Montgomery Modular Multiplier (referred to as pMMM), which is built upon *n*-bit pipelined multiplier-accumulators utilizing DSP primitives as digit multipliers. To support the high-speed use, we modify the modular adder/subtractor in [11] to support pipelining, which here is referred to as pipelined Modular Adder/Subtractor (pMAS). Additionally, we adapt the Montgomery ladder algorithm recently presented by Hamburg [22], which to date, provides the most efficient computation. Moreover, we managed to employ a more efficient scheduling compared to the original approach, in which we eliminate the use of an additional temporary register. Tested in the 7-series FPGA (i.e., Virtex-7, Kintex-7, and XC7Z020), our method yields the latency of 0.139, 0.138, and 0.206 ms, respectively, which to the best of our knowledge, is the fastest in literature for generic curves. In fact, even when compared to the methods that use special prime forms (e.g., [23,24,25,26], which take 0.054, 0.101, 0.400, and 0.620 ms, respectively), our approach is still considerably competitive.

Apart from speed, another advantage of our approach is that it can work for arbitrary prime modulus. Hence, multi-curve parameters can be provided in a single, unified ECC processor. This will be very beneficial in the real-life cases, as previously discussed. Lastly, we also aim to minimize the risk of side-channel attacks (SCA), in which adversaries may extract secret key information without breaking the primitives by analyzing the variations of timing (i.e., timing attack), power consumption (i.e., differential power analysis attack (DPA)), and electromagnetic emission (i.e., EM attack)) of the cryptographic device [27]. Our architecture performs all the underlying operations invariant for any key value, executing the ECPM in a fully constant manner. This includes utilizing Fermat’s little theorem for the field inversion operation instead of extended Euclidean as the more commonly used algorithm. Thus, SCA-resistant property can be preserved [28].

The contributions of this paper can be summarized as follows:

1. We propose a high-speed, unified ECC processor that is generic for arbitrary prime modulus on Weierstrass curves. To the best of our knowledge, in terms of generic implementation, it is the fastest among the existing literature.
2. For the underlying architecture, we propose a novel and fast pipelined Montgomery Modular Multiplier (pMMM), which is constructed from an *n*-bit pipelined multiplier-accumulator. The speed-up comes from combining two existing multiplication algorithms: schoolbook long and Karatsuba–Ofman multiplications, enabling parallelization of digit multiplications while preserving low complexity. Moreover, to further optimize the process, we utilize DSP cores as digit multipliers, resulting in a higher speed multiplier compared to other existing methods.
3. To balance the speed of our fast pMMM, we also propose a unified and pipelined Modular Adder/Subtractor (pMAS) for the underlying field arithmetic operations. In particular, we modify the modular adder/subtractor in [11] to support pipelining, and employ an adjustable radix. The proposed design offers better flexibility in adjusting the performance of the ECC processor.
4. Additionally, we propose a more efficient and compact scheduling of the Montgomery ladder for the algorithm for ECPM in [22], in which our implementation does not require any additional temporary register as opposed to one additional register in the original algorithm. As a result, it only needs 97 clock cycles to perform ladder operation per bit scalar (for 256-bit size).
5. Since our ECC processor and the underlying field multiplier (i.e., pMMM) are generic for arbitrary prime modulus, we can support multi-curve parameters in a single ECC processor, forming a unified ECC architecture.
6. Lastly, our architecture performs the ECPM in constant time by employing a time-invariant algorithm for each module, including using Fermat’s little theorem to carry out field inversion, making the algorithm secure against side-channel attacks.

The remainder of this paper is organized as follows. We provide several preliminaries in Section 2 before moving on to the detail of our proposed ECC architecture in Section 3. In Section 4, we present our result of hardware implementation and the discussions regarding its comparison to the existing methods. Lastly, Section 5 concludes the paper.

## 2. Preliminaries

### 2.1. Hamburg’s Formula for ECPM with Montgomery Ladder

An elliptic curve over a prime field GF(*p*) is defined by the coordinates (x,y) that satisfies the short Weierstrass equation as follows:

(1) $$y^{2}=x^{3}+ax^{2}+b\;\mathrm{mod}\;p$$

where *a* and *b* satisfy $4a^{3}+27b^{2}\neq 0$ to avoid singularity on the curve.

The Montgomery ladder [29] is a general algorithm for computing the power or scalar multiple of points, which is considered resistant against side-channel attacks due to its constant-time operation. Let *k* be a scalar and $P=(x_{P},y_{P})$ be a point in an elliptic curve *E*. An elliptic curve point multiplication (ECPM) Q=kP is the repeated addition of point *P* (i.e., P+P+P+…+P) for *k*-times. This operation can be performed using the Montgomery ladder, which generally consists of point addition and point doubling operations. In 2020, Hamburg [22] proposed an improved Montgomery ladder formula for ECPM that reduces the number of arithmetic operations in the ladder algorithm to as low as eleven multiplications and eight additions. This formula allows four multiplications and three additions to be performed in parallel. To date, this algorithm is considered as the state-of-the-art for the Weierstrass curve.

Let Equation (2) be the initial state of the Montgomery ladder for an elliptic curve in the short Weierstrass equation as previously shown in Equation (1).

(2) $$P=\left(x_{P},y_{P}\right),Q:=\left(x_{Q},y_{Q}\right),R:=P+Q:=\left(x_{R},y_{R}\right)$$

A single step of the ladder operation calculates:

(3) $$P=\left(x_{P},y_{P}\right),S:=Q+R=\left(x_{S},y_{S}\right),T:=2R=\left(x_{T},y_{T}\right)$$

The ladder operation from Equations (2) and (3) can be calculated using Algorithm 1. Before performing ladder operation, the input $P=(x_{P},y_{P})$ is encoded into Hamburg’s ladder state ($X_{QP}$, $X_{RP}$, *G*, $Y_{Q}$, $Y_{R}$), here referred to as the ladder setup. Accordingly, at the final step, the ladder state is decoded back to $Q=(x_{Q},y_{Q})$, which is the ECPM result in the affine coordinate. Consequently, the complete Montgomery ladder algorithm for ECPM with Hamburg’s formula is given in Algorithm 2. Note that since the initial state of the ladder calculates $\left(Q,R\right)\leftarrow \left(P_{0},2P_{0}\right)$, which requires the most significant bit (MSB) of input scalar *k* to be 1, the input scalar is rewritten by adding a multiple of *q*.
**Algorithm 1** Hamburg’s Montgomery Ladder Formula [22].**Input:**$\left(X_{QP},X_{RP},Y_{Q},Y_{R},G\right)$**Output:**$\left(X_{SP},X_{TP},Y_{S},Y_{T},G^{\prime }\right)$1:$X_{QP}^{\prime }=X_{QP}\times G$2:$X_{RP}^{\prime }=X_{RP}\times G$3:$L=Y_{Q}\times Y_{R}$4:$H=Y_{R}^{2}$5:$J=X_{RP}^{\prime }-L$6:$M=J+X_{RP}^{\prime }-H$7:$X_{SP}=H\times L$8:$V=H\times (X_{QP}^{\prime }-L$)9:$X_{TS}=X_{RP}^{\prime }\times J+V$10:$Y_{S}=\left(J\times L+V\right)\times H$11:$Y_{T}=M\times X_{TS}+Y_{S}$12:$G^{\prime }=X_{TS}^{2}$**Algorithm 2** Montgomery Ladder.**Input:**$k,q\leq 2^{n},P\in E\left(F_{p}\right)$ **Rewrite**$k\leftarrow 2^{n}+\left(k-2^{n}\;\mathrm{mod}\;q\right)$ **Output:**Q=kP1:$\left(X_{QP},X_{RP},Y_{Q},Y_{R},G\right)\leftarrow LADDER\_SETUP\left(x_{P},y_{P}\right)$2:**for**i=n−1 to 0 **do**3:    **if** $k_{i}$ **then**4:        $\left(X_{QP},X_{RP},Y_{Q},Y_{R},G\right)\leftarrow LADDER\_UPDATE\left(X_{QP},X_{RP},Y_{Q},Y_{R},G\right)$5:    **else**6:        $\left(X_{RP},X_{QP},Y_{R},Y_{Q},G\right)\leftarrow LADDER\_UPDATE\left(X_{RP},X_{QP},Y_{R},Y_{Q},G\right)$7:$\left(x_{Q},y_{Q}\right)\leftarrow LADDER\_FINISH\left(X_{RP},X_{QP},Y_{R},Y_{Q},G\right)$8:**return**$\left(x_{Q},y_{Q}\right)$

#### 2.1.1. Ladder Setup

Essentially, the ladder setup calculates $R=2P_{0}$, which is the point doubling operation. To eliminate the costly field inversions in the ladder operation, Jacobian projective coordinates are generally used; in our case, we use $Z=2y_{P}$, giving the ladder setup formulas as presented in Equations (4)–(7).

(4) $$\begin{array}{cc} M & =\frac{3x_{P}^{2}+a}{2y_{P}}Z=3x_{P}^{2}+a \end{array}$$

(5) $$\begin{array}{cc} X_{RP} & =\left(x_{R}-x_{P}\right)Z^{2}=M^{2}-3x_{P}Z^{2} \end{array}$$

(6) $$\begin{array}{cc} Y_{R} & =2MX_{RP}+Y_{P} \end{array}$$

(7) $$\begin{array}{cc} G & ={\left(x_{R}-x_{Q}\right)}^{2}Z^{4}=X_{RP}^{2} \end{array}$$

Note that since Q=P, then $X_{QP}=\left(x_{Q}-x_{P}\right)Z^{2}=0$ and $Y_{Q}=Y_{P}=2y_{P}Z^{3}=Z^{4}$.

#### 2.1.2. Ladder Final

In order to complete the ladder operation, the final $x_{Q}$ and $y_{Q}$ must be recovered from the ladder state, as shown in Equations (8)–(10).

(8) $$\begin{array}{cc} Y_{P} & =Y_{R}-MX_{RP} \end{array}$$

(9) $$\begin{array}{cc} \frac{1}{Z} & =\frac{2y_{P}\left(M^{2}-X_{QP}-X_{RP}\right)}{3x_{P}Y_{P}} \end{array}$$

By calculating 1/Z from Equation (9), we obtain Equation (10).

(10) $$\begin{array}{cc} \left(x_{Q},y_{Q}\right) & =\left(\frac{X_{QP}}{Z^{2}}+x_{P},\frac{Y_{Q}}{2Z^{3}}\right) \end{array}$$

### 2.2. Montgomery Modular Multiplication

Montgomery modular multiplication [30] is an efficient method for modular multiplication proposed by Peter L. Montgomery in 1985, which operates without any trial divisions by transforming the number into a special form such that the dividend is always a multiple of the divisor. Let R>P with gcd(R,P)=1. The Montgomery multiplication calculates $ABR^{-1}\;\mathrm{mod}\;P$ with 0≤AB<RP. Algorithm 3 shows a constant-time implementation of the Montgomery modular multiplication. Since *n*-bit *P* is an odd modulus, we can take $R=2^{n}$, which results in an easy division by shifting. Montgomery multiplication requires the number to be transformed into the Montgomery domain. However, the transformation is performed only once when used with many intermediate multiplications in the algorithm (e.g., ECPM).
**Algorithm 3** Montgomery Multiplication.**Input:** an odd modulus *p* of *n*-bits, $R=2^{n}$, gcd(R,p)=1             M=−modR,             A,B:A,B<p<R **Output:**
$ABR^{-1}\;\mathrm{mod}\;p$1: x←AB▹ 1st multiplication2: s←(xmodR)MmodR▹ 2nd multiplication 3: t←(x+sp)/R▹ 3rd multiplication4: u←t−p▹ subtraction5: **if**
u<0
**then**▹ MSB of *u*6:       **return**
*t* 7: **else** 8:       **return**
*u*


## 3. Proposed Architecture

This section presents the proposed generic hardware architecture for high-speed ECC processors over GF(p). Since the performance of ECC processors mostly depends on the underlying modular multiplication, our proposed architecture focuses on optimizing the modular multiplier module, mainly to reduce the latency of multiplication as well as the number of multiplication for each Montgomery ladder step. Moreover, for further optimization, we adopt the modular adder/subtractor first introduced in [11], then modify it to support pipelining, which yields even higher speed performance.

First, to realize a generic ECC architecture, we employ the Montgomery modular multiplier, which does not require any special prime form. Although this approach tends to be slower, it offers much greater flexibility when dealing with various curve parameters. Montgomery multiplication does require the input operands to be transformed into the Montgomery form. The conversions are performed twice: at the beginning (i.e., before the multiplication), and at the end to convert the number back to its original form. Nevertheless, the cost of conversion is negligible compared to the advantage of the execution in the Montgomery domain.

Furthermore, to achieve a high-performance ECC processor, we propose an *n*-bit pipelined Montgomery Modular Multiplier (pMMM), which is essentially constructed from *n*-bit pipelined multipliers and the corresponding Montgomery reduction circuit. The calculation for Montgomery reduction is presented in Algorithm 3, whereas the modular multiplication is performed via three multiplications and one subtraction, executed in sequence while interleaved with other pMMM threads. In our FPGA implementation, the *n*-bit pipelined multiplier-accumulator is mainly constructed from DSP primitives as digit multipliers.

Consequently, to match the speed of pMMM when performing the point multiplication (i.e., ECPM), we also propose a fully pipelined Modular Adder/Subtractor (pMAS), which offers better flexibility in adjusting the performance of the ECC processor (e.g., maximum frequency and latency).

We also implement the Montgomery ladder algorithm for ECPM by Hamburg [22], which is complete (i.e., works on any input point and scalar), and thus, can work on generic Weierstrass curve over GF(p). Furthermore, to date, [22] offers the most efficient computation among other existing algorithms. By utilizing this algorithm, we can unify the construction for multiple curves into a single-core ECC processor.

Furthermore, we managed to yield a slight improvement from [22] in our implementation. Instead of utilizing six registers as presented in [22], our compact and efficient scheduling reduces the need to only five, without any additional temporary registers. This is achieved by interleaving four modular multiplications using pMMM and *d*-stage pMAS.

In terms of the field inversion, we employ Fermat’s little theorem to preserve the SCA-resistant property by performing the inversion in constant time. This approach also does not require a separate module because the inversion computation, which essentially is exponentiation, is also carried out by pMMM.

### 3.1. Pipelined Montgomery Modular Multiplication (pMMM)

#### 3.1.1. Overview of pMMM

Modular multiplication is the most extensive arithmetic operation in an ECC processor, which heavily affects the performance and the occupied area of the processor. Our proposed approach, namely the pipelined Montgomery Modular Multiplication (pMMM), can process multiple input operands. The pipelined architecture of pMMM enables the sequence of multiplications to be executed concurrently, hence sharing the same resources. Additionally, the heart of pMMM is a multiplier that supports pipelining as well, enabling a greater speed-up in the computation. In the following subsection, we will first go into the detail of our proposed pipelined multiplier-accumulator before discussing the general architecture of the pMMM.

#### 3.1.2. Proposed Pipelined Multiplier-Accumulator

Our pipelined multiplier-accumulator is essentially a combination of schoolbook long multiplication and Karatsuba–Ofman multiplication algorithm [21]. Schoolbook long multiplication is a naïve way to perform multiplication with $n^{2}$ complexity, where *n* is the number of digits. Even though it has a relatively high complexity, all the digit multiplications can be executed in parallel. Furthermore, it supports high-performance hardware implementation by adopting the divide-and-conquer method. On the other hand, Karatsuba–Ofman multiplication offers lower complexity but with the trade-off that it is difficult for parallelization due to its recursive approach when dealing with higher bit length. We have managed to find a better approach by combining both algorithms to support multiplication in parallel while retaining the small complexity.

The mathematical formulation for our algorithm is as presented in Equations (11)–(14). Let *a* and *b* be the two *n*-bit numbers to be multiplied, α be the chosen radix, whereas i,j, and *k* be the indices. A general schoolbook long multiplication (Equation (11)) can be split into two terms: by certain index *k*, that is when j=i; and when j≠i, as shown in Equation (12). The derivation to Equation (14) shows that the second term is, in fact, a Karatsuba–Ofman multiplication method while the first term remains the schoolbook long multiplication formula. Utilizing the property of schoolbook long multiplication, which can be run in parallel since there is no dependency to the previous nor the succeeding computation, while also reducing the length of multiplication by employing the Karatsuba–Ofman method, a significant gain in speed can be acquired. To be exact, the time complexity is reduced to $\frac{1}{2}\left(n^{2}+n\right)$ from $n^{2}$ in the original schoolbook case. Compared to Karatsuba–Ofman, our algorithm indeed is higher in complexity, but with the significant advantage of parallelization for the hardware implementation.

(11) $$\begin{array}{cc} ab & =\sum_{i=0}^{m-1}\sum_{j=0}^{m-1}a_{i}b_{j}\alpha^{\left(i+j\right)} \end{array}$$

(12) $$\begin{array}{cc} \; & =\sum_{k=0}^{m-1}a_{k}b_{k}\alpha^{2k}+\sum_{i=0}^{m-1}\sum_{j=0,j\neq i}^{m-1}a_{i}b_{j}\alpha^{\left(i+j\right)} \end{array}$$

(13) $$\begin{array}{cc} \; & =\sum_{k=0}^{m-1}a_{k}b_{k}\alpha^{2k}+\sum_{i=0}^{m-1}\sum_{j=i+1}^{m-1}\left[a_{i}b_{j}+a_{j}b_{i}\right]\alpha^{\left(i+j\right)} \end{array}$$

(14) $$\begin{array}{cc} \; & =\sum_{k=0}^{m-1}a_{k}b_{k}\alpha^{2k}+\sum_{i=0}^{m-1}\sum_{j=i+1}^{m-1}\left[\left(a_{i}+a_{j}\right)\left(b_{i}+b_{j}\right)-a_{i}b_{i}-a_{j}b_{j}\right]\alpha^{\left(i+j\right)} \end{array}$$

In terms of the hardware implementation, the speed increase in our approach is mainly contributed by the digital signal processor (DSP) cores in the modern FPGA that function as digit multipliers. The proposed multiplier is fully pipelined, in which new input can be processed for each cycle. The divide-and-conquer method employed in the schoolbook long multiplication is adopted, but each digit is optimized with Karatsuba-Ofman multiplication, which is later assembled with the compression module, the Carry Save Adder Tree (CSAT). All ripple-carry adders (RCAs) used in the multiplier module are implemented using a fast carry chain in modern FPGA. This primitive works in conjunction with Lookup Tables (LUTs) to construct the adders [31].

**Figure 1:**
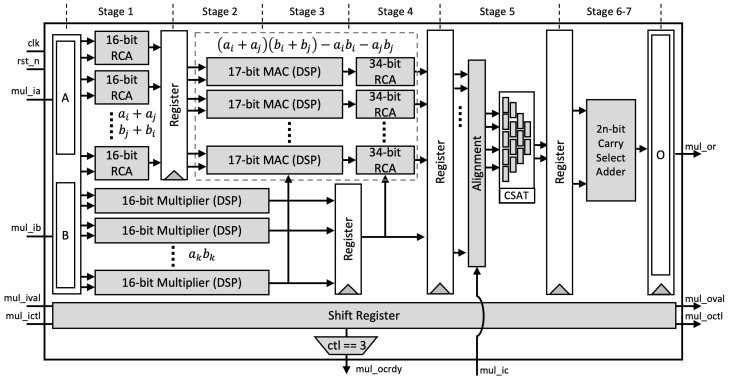
Proposed pipelined multiplier-accumulator.

Equation (14) is implemented as an 8-stages pipeline, shown in Figure 1, as described below.

**Figure 2:**
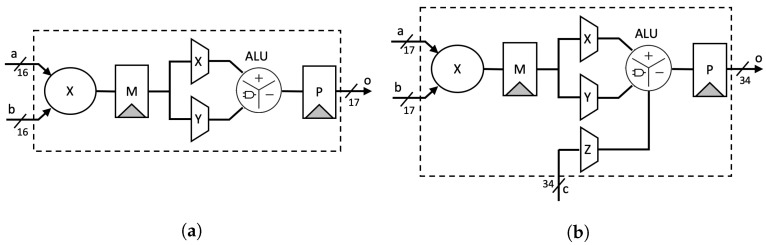
Digital signal processor (DSP) utilization setup: (**a**) 16-bit multiplier; (**b**) 17-bit multiply-accumulate.

**Figure 3:**
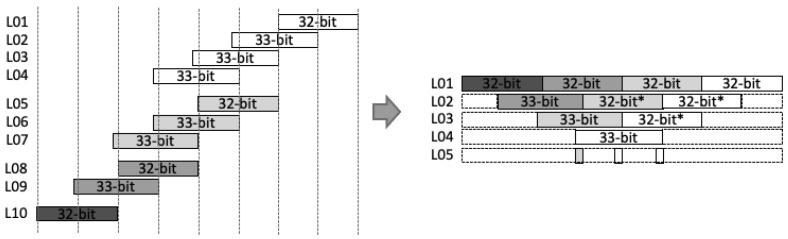
Example of alignment for intermediate values in a 64-bit multiplier.

**Figure 4:**
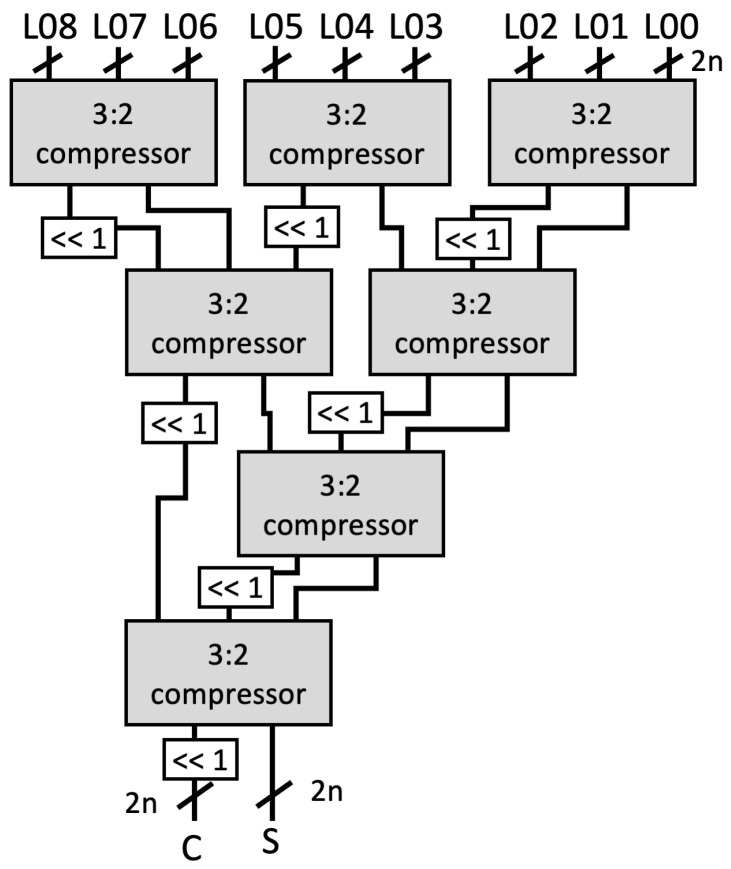
Example of Carry Save Adder Tree (CSAT) for nine inputs.

- Stage-1: Two inputs *A* and *B* are split based on the radix (digit size), which is into 16 bits in our design. Afterward, a parallel 16-bit RCA is used to compute $a_{i}+a_{j}$ and $b_{j}+b_{i}$. At the same time, parallel DSP cores are utilized as 16-bit digit multipliers to compute $a_{k}b_{k}$. As shown in Figure 2a, we employ a two-stage pipeline for the DSP cores to achieve better performance, as recommended in [32].
- Stage-2: We again utilize the DSP cores as a 17-bit Multiply-Accumulate (MAC) function to compute the Karatsuba–Ofman multiplication, $\left(a_{i}+a_{j}\right)\left(b_{i}+b_{j}\right)-a_{i}b_{i}$. $\left(a_{i}+a_{j}\right)$ and $\left(b_{i}+b_{j}\right)$ are obtained from the output of RCAs at the first stage, as shown in Figure 2b.
- Stage-3: The outputs of 16-bit multipliers $a_{k}b_{k}$ are routed to the input accumulator in the MAC modules as $a_{i}b_{i}$.
- Stage-4: The final accumulation for Karatsuba–Ofman is computed by a 34-bit RCA. The equation $\left(a_{i}+a_{j}\right)\left(b_{i}+b_{j}\right)-a_{i}b_{i}-a_{j}b_{j}$ results in a 33-bit length. At this stage, mul_ocrdy is set when the CTL value is 3. It means that the input mul_ic is ready to be included in the CSAT at Stage 5 as the final accumulation of the Montgomery reduction algorithm. The algorithm itself is as presented in Algorithm 3.
- Stage-5: Before being processed by the CSAT, all intermediate values are aligned to reduce the number of inputs in CSAT as well as the depth of the tree. This is due to the additional bit length on each intermediate value, i.e., 33-bit instead of 32-bit length. Figure 3 shows the example of the alignment process for four-input CSAT.
  All aligned intermediate values, including the input mul_ic, are assembled by CSAT where the compressor components in the CSA use LUT6_2, a similar 3:2 compressor circuit proposed by [11]. However, while they use multiple compressor circuits (e.g., a 4:2 compressor in [11]) to construct the multiplier, we employ the homogeneous 3:2 compressor to achieve a balanced performance, as illustrated on Figure 4.
- Stage-6 and 7: The sum and carry as the outputs of CSAT are then fed to the carry-select adder to obtain the final product. Note that we use the carry-select adder proposed by Nguyen et al. [33] due to its relatively short delay propagation. In the carry-select adder by [33], both options for the carry are computed. Subsequently, the carry is solved similarly to that of the carry-lookahead adder (CLA). Lastly, the sum output is then generated with the final carry for each bit [34].
- Stage-8: A register is used to hold the output mul_or. The outputs o_val and o_ctl are given with respect to the input values i_val and i_ctl, respectively, which are shifted through the stages via a shift register.

#### 3.1.3. Montgomery Modular Multiplication Using pMMM

**Figure 5:**
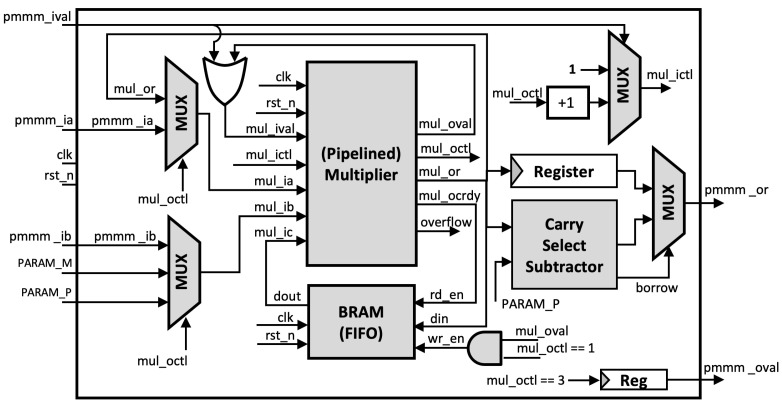
Proposed Pipelined Montgomery Modular Multiplier (pMMM).

In our pMMM architecture as shown in Figure 5, a single execution of Montgomery modular multiplier consists of three steps of multiplications and one step of subtraction, divided into four steps as follows:

1. The pMMM starts by multiplying the *n*-bit inputs pmmm_ia and pmmm_ib, resulting in a 2n-bit product, which is then stored in the first-in, first-out (FIFO) buffer. This product will be used later in the third multiplication. Note that our FIFO buffer uses block RAM (BRAM) to reduce the required number of registers, where the depth of the FIFO buffer depends on the number of possible multiplication processes that can be executed concurrently.
2. The *n*-bit LSB product of Step 1 is multiplied with the precalculated constant PARAM_M.
3. Accordingly, the *n*-bit LSB product of Step 2 is multiplied by the modulus PARAM_P. In this multiplier, the product that was previously stored in the FIFO at Stage 1 is used as the input mul_ic to be included in CSAT in the multiplier module. This gives the benefit that we do not need to make additional 2n-bit adders. Instead, we include it in the CSAT.
4. The *n*-bit MSB of the third multiplication product is then evaluated and corrected using the carry-select subtractor, so that the output of pMMM is within the range [0, *P*].

**Figure 6:**
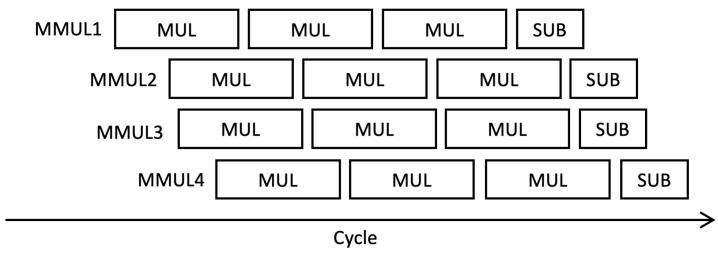
Example of scheduling for four pipelined Montgomery Modular multiplier (pMMM) processes.

Since the multiplier can be pipelined, the input operand for pMMM can as well be pipelined. In particular, we support up to eight pipelined multiplications, in accordance with the number of pipeline stages of our multiplier. Each execution in a single pMMM operation is controlled by CTL, which is propagated during pMMM execution and incremented for each step. However, in our case, Hamburg’s formula for Montgomery ladder, as previously discussed in Algorithm 1, can only be performed up to four multiplications concurrently. Therefore, we adjust the FIFO depth to four, with a data width of 2n. Each pMMM operation does not need to be executed in sequence next to each other in one cycle, yet it can be performed even if there is a delay step between input operand. However, all sequences must fit in eight clock cycles and can be used again after the first pMMM output is received. This is done to ensure that no internal steps of pMMMs are in conflict. The full sequence of multiple inputs of pMMMs is illustrated in Figure 6.

### 3.2. Pipelined Modular Adder/Subtractor (pMAS)

Modular addition and subtraction operations also play a significant role in an ECC architecture, which also affect the processor’s performance. The authors of [11] propose a unified 64-bit modular adder/subtractor that is designed to work with redundant numbers. However, their design can not be pipelined and uses a shift register to compute modular adder for higher bit length. In this paper, in order to match the speed of our multiplier, we introduce the pipelined version of the modular adder/subtractor in [11], which is also able to operate as a modular adder or subtractor by specifying the input i_op. Furthermore, instead of fixing the radix to a 64-bit operand, the radix in our design can be adjusted by specifying the number of stage *d*. Thus, the performance of our modular adder/subtractor can be adjusted depending on the requirement and available hardware resources. We refer to our architecture as the pipelined modular adder/subtractor (pMAS).

Let *d* be the number of pipeline stages and *m* be the radix size. Each pipeline stage takes *m*-bit input operand, as shown in (15). An *m*-bit ripple-carry adder/subtractor is implemented on each stage as the building block of pMAS.

(15) $$m=\left\lfloor \frac{n}{d}\right\rfloor$$

**Figure 7:**
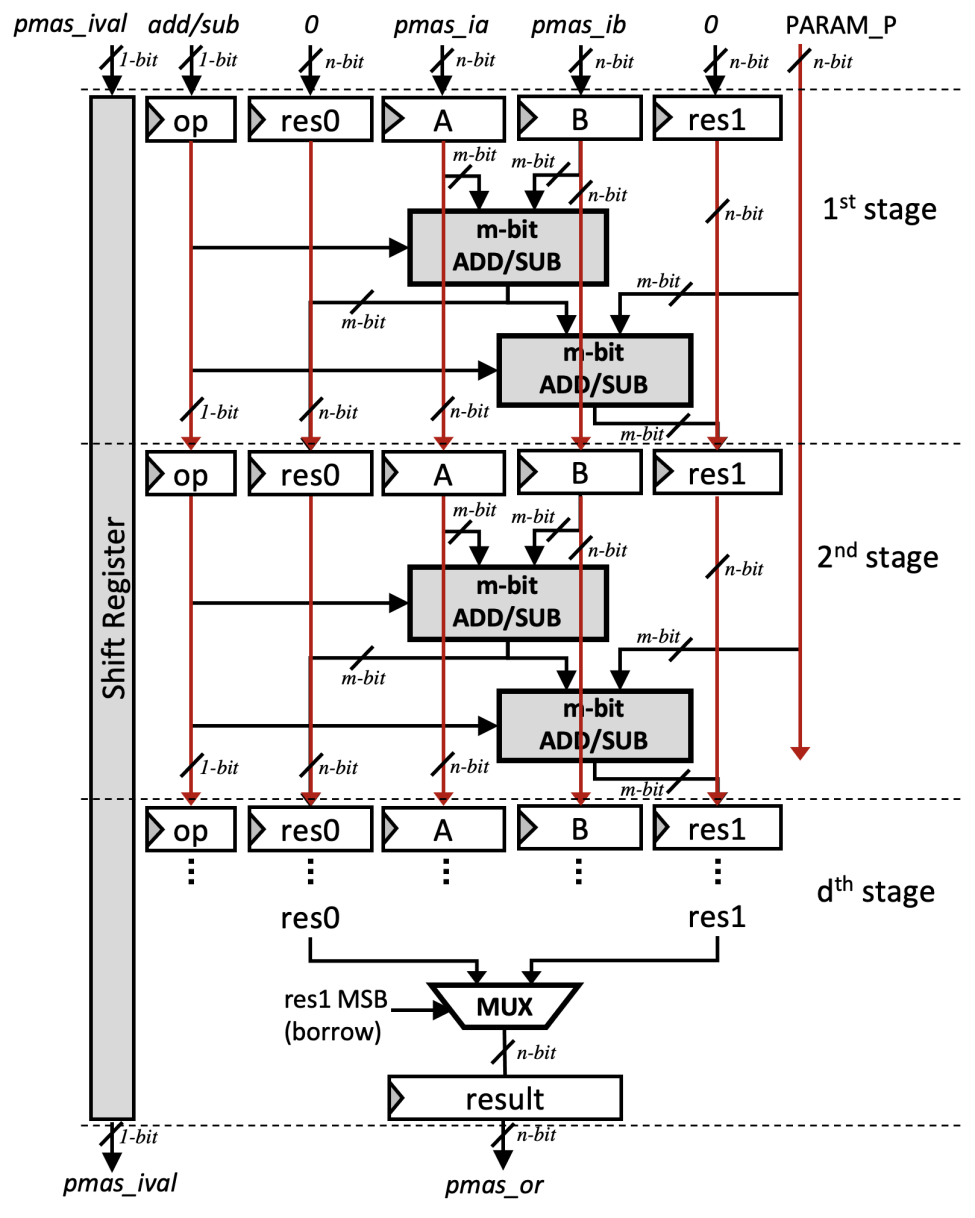
Proposed Modular adder/subtractor (pMAS).

Our pMAS is performed in constant time. As shown in Figure 7, computation of both a±b and a±b±p are performed simultaneously whenever arbitrary input is received so that the secret values cannot be retrieved using power and timing analysis.

### 3.3. Modular Inversion Implementation

In order to be a fully constant-time ECPM, we use the modular inversion based on Fermat’s little theorem rather than the binary extended Euclidean algorithm. In summary, the theorem states that if *p* is a prime number and *a* is any number not divisible by *p*, then it satisfies Equation (16) [35].

(16) $$a^{p-1}\equiv 1\left(\mathrm{mod}\;p\right)$$

By multiplying both sides with $a^{-1}$, we obtain Equation (17), which infers that an inversion can be accomplished by utilizing exponentiation.

(17) $$a^{-1}\equiv a^{p-2}\left(\mathrm{mod}\;p\right)$$

The inversion can be easily performed by using the Montgomery ladder for exponentiation [29], which is also SCA-resistant due to its characteristic of constant-time operation. However, many proposals refrain from leveraging Fermat’s little theorem for modular inversion due to the extensive use of multiplications (i.e., 2n multiplications to achieve an exponentiation). Nevertheless, in our case, the hardware implementation of Fermat’s little theorem still gives a competitive advantage by incorporating pMMM, yielding a relatively fast implementation via concurrent execution of two modular multiplications (i.e., $a_{1}a_{2}$ and $a_{1}^{2}$ or $a_{2}^{2}$ in Algorithm 4). Furthermore, no additional module for inversion is required, which directly reduces the slice overhead.
**Algorithm 4** Constant-time Field Inversion algorithm     **Input:** *a* and prime modulus *p* of *n*-bits, 0≤a<p     **Output:** $a^{-1}\;\mathrm{mod}\;p$1:**procedure**FieldInverse(a,p)2:    e=p−23:    $a_{1}$ = *a*, $a_{2}$ = $a^{2}$4:    **for** i=n−2 to 0 **do**5:        **if** $e_{i}$ = 0 **then**6:               $a_{2}$ = $a_{1}a_{2}$, $a_{1}$ = $a_{1}^{2}$7:        **else**8:               $a_{1}$ = $a_{1}a_{2}$, $a_{2}$ = $a_{2}^{2}$            9:    **return** $a_{1}$

### 3.4. Montgomery Ladder Scheduling

**Figure 8:**
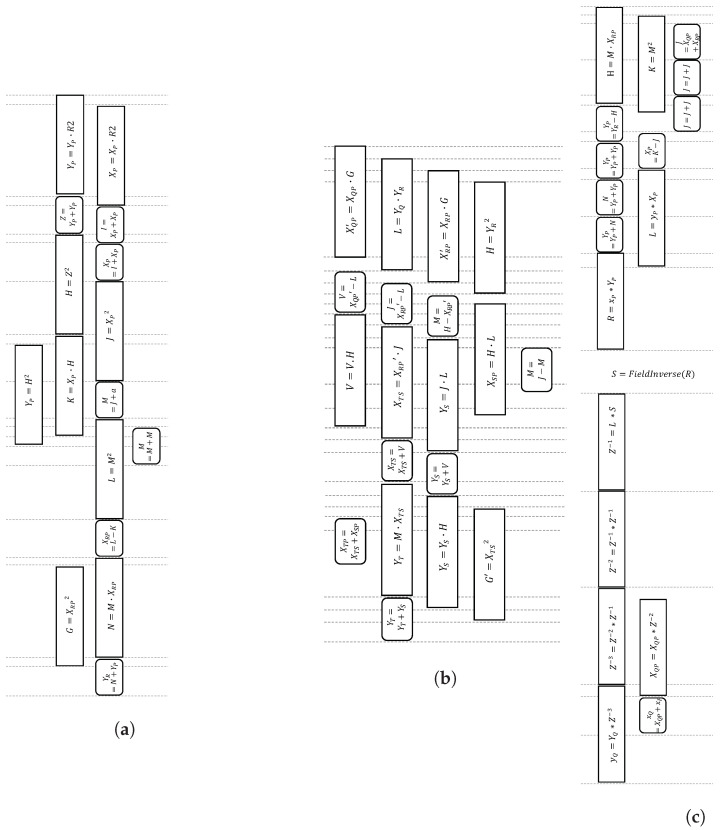
Proposed scheduling for Montgomery (**a**) ladder setup; (**b**) ladder update; and (**c**) ladder final.

The improved Montgomery ladder formula by [22], as depicted in Algorithm 1, incurs eleven multiplications and eight additions, and allows parallelization up to four multiplications and three additions per bit scalar. To date, this latest algorithm is considered the fastest for the Weierstrass curve. We adopt and optimize the scheduling of this algorithm by incorporating pMMM and pMAS in the ladder update (Algorithm 1), as well as the ladder setup (Equations (4)–(7)) and ladder final (Equations (8)–(10)), as presented in Figure 8. Up to four modular multiplications and modular adder/subtractors can be pipelined, making a compact scheduling process. Moreover, our proposed scheduling does not require any additional registers, as opposed to the original approach in [22], which requires a temporary register.

Note that a complete ECPM algorithm, as illustrated in Algorithm 2, includes ladder setup and ladder finish. Ladder update is the part that severely contributes to the latency of the circuit since it is executed iteratively per bit scalar.

### 3.5. Generic ECC Architecture

The main building blocks of ECC processors are pMMM and pMAS, which play a major role in improving the speed of ECPM. The use of pMMM eliminates the restriction of modulus to the special prime form, making our ECC architecture generic for arbitrary prime modulus. The modular inversion uses Fermat’s little theorem, which also exploits the use of pMMM, making the algorithm fast even with an extensive number of multiplications. pMMM enables the modular inversion implementation without any additional modules.

**Figure 9:**
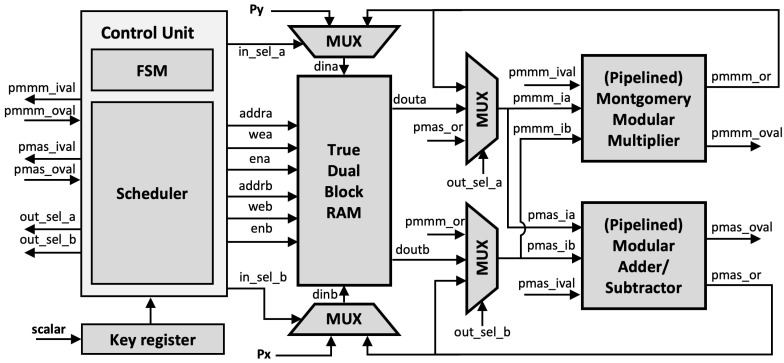
Proposed elliptic curve cryptography (ECC) architecture.

The proposed generic ECC architecture is shown in Figure 9. In addition to the pMMM and pMAS module, True Dual Port (TDP) RAM is implemented using BRAMs, which reduces the slice overhead. All operands and constants are stored in the TDP RAM.

The Montgomery ladder, as illustrated in Algorithm 2, requires conditional swap for $X_{QP}↔X_{RP}$ and $Y_{Q}↔Y_{R}$ depending on the scalar bit, which may pose a security risk of a side-channel leakage. However, the benefit of using BRAM is that it indirectly preserves side-channel resistance since the actual swap is applied to the operand address instead of the operand values, which is a few bits length. Thus, the ECPM with our proposed architecture is performed in constant time and does not have any scalar-dependent branches.

Since both pMMM and pMAS use registers to hold the output values, the intermediate result can be fed back to its input instead of being stored in TDP RAM, making the execution faster and allowing efficient utilization of the BRAM. Additionally, the multiplexer is connected to each input operand so that it can provide the input depending on the ladder scheduling.

#### Unified Architecture

**Figure 10:**
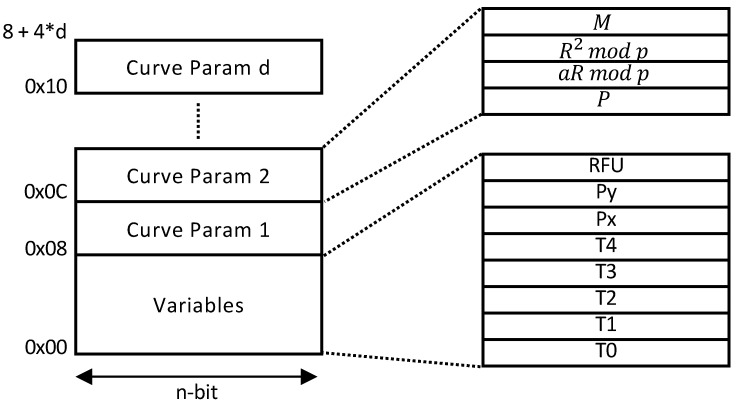
Address map.

Our architecture also supports multi-curve parameters in a single ECC processor. The architecture in Figure 9 can be transformed into a unified architecture since pMMM and pMAS do not restrict to any modulus value or form. However, a few modifications are required in the pMMM modules. In particular, the input and output of the third multiplication in Algorithm 3 require to be sliced, depending on the modulus size. This can be done by implementing a multiplexer to both input and output of pMMM at Step 3. Nevertheless, other components in the architecture remain the same. Additionally, since the curve domain parameters are stored in the BRAM, extending the support to different curve parameters will only increase the BRAM depth without affecting other modules (e.g., pMMMs, pMASs). The address map is shown in Figure 10.

## 4. Hardware Implementation Result and Discussion

Our proposed design has been described by SystemVerilog HDL. Synthesizing, mapping, placing, and routing were carried out using Xilinx Vivado 2020, targeting three modern devices: Xilinx Virtex-7 (XC7VX690T), Kintex-7 (XC7K325T), and Zynq (XC7Z020) FPGA, for a more comprehensive evaluation and a thorough comparison with other recent works that use the 7-series FPGA.

### 4.1. Result and Analysis of Generic Implementation on Weierstrass Curve

The result of our generic ECC implementation as well as several related papers on the Weierstrass curve are presented in Table 1. In our case, we achieve the fastest speed among other proposals for 256-bit modulus size, with 0.139, 0.138, and 0.206 ms on Virtex-7, Kintex-7, and Zynq, respectively. Our fastest implementation (Virtex-7) requires 6909 slices, while Kintex-7 and Zynq utilize a slightly higher number of slices (7115 and 7077). On all of the three platforms, we utilize 136 DSPs and 15 BRAMs. As can be inferred from the table, our architecture yields the highest performance in terms of execution time compared to other existing techniques. This can be achieved due to the fact that our implementation requires lower clock cycles. In detail, the performance of each arithmetic and ladder operation for Kintex-7 is presented in Table 2.

Prior to our work, the implementation with the fastest speed is the proposal by Ma et al. [16] in 2014, which gives the execution time of 0.380 ms. It also achieves a considerably high maximum frequency of 291 MHz and consumes a relatively low resource of 1725 slices and 37 DSPs. The speed mainly comes from their quotient pipelined Montgomery multiplier combined with a parallel array design. However, since they run on an older platform (i.e., Virtex-5), it is not comparable to our result.

To the best of our knowledge, the state-of-the-art generic ECC processor for high-speed implementation in the 7-series FPGA is the method by Roy et al. [11]. Their technique is primarily intended for the Montgomery curve, but since their proposed method focuses on implementing the Montgomery multiplier, they also extend their implementation to short Weierstrass curves and provide the performance analysis of their approach. In particular, they require eight dual multiplications and three single additions to perform one Montgomery ladder iteration.

In comparison to the method in [11] for the same target device (i.e., XC7Z020 FPGA), our approach yields an execution time of 0.139 ms whereas [11] requires 0.459 ms for a single ECPM execution. In other words, our method is approximately three times faster. However, readers may notice from Table 1 that in terms of the maximum frequency, the implementation in [11] reaches a higher value of 208.3 MHz while ours is 156.8 MHz. Nevertheless, since our method employs fewer clock cycles (i.e., 32.3k cycles as opposed to 95.5k), our overall speed outperforms their proposed approach.

In terms of the area overhead, our implementation indeed requires a relatively larger area compared to the existing proposed methods. It requires a higher number of hard IPs (i.e., DSP and BRAM). However, from the time/area efficiency perspective, as shown in Table 1, the cost of our method is relatively similar to the existing 7-series implementations. Note that the time–area is calculated from the occupied slices only. Furthermore, modern devices available in the market (i.e., Virtex-7, Kintex-7) are generally equipped with a relatively large resource. In fact, from the hardware utilization perspective, as presented in Table 3, the overall architecture only utilizes below seven percent of the total area in the FPGA. Hence, our high-speed architecture would still be greatly suited for services requiring low latency (speed-critical applications), such as for runtime authentication in automated vehicles, web server certification, etc. [11].

Regarding other proposals in the 7-series FPGA implementation, Bajard et al. [15] proposed a residue number system (RNS)-based ECC processor that utilizes Cox–Rower architecture for fast parallel Montgomery multiplication, which was initially introduced by [36]. They introduce a new ALU design utilizing the second level of Montgomery reduction within each RNS unit, increasing the maximum working frequency compared to the original one. On Kintex-7, they consume 1630 slices, 46 DSP cores, and 16 BRAMs, operating at 281.5 MHz maximum frequency, with a latency of 0.612 ms for a 256-bit ECPM.

Asif et al. [14] proposed a residue number system (RNS)-based ECC processor that utilizes a serial-parallel approach for its modular reduction to balance its time and area performance. With the hardware utilization of 18.8k LUTs, their method achieves 86.6 MHz maximum frequency and a relatively larger latency compared to other recent approaches.

On the earlier platform, Shah et al. [17] proposed a redundant-signed-digit (RSD)-based ECC processor leveraging Montgomery multiplier that uses parallel computation technique operating in (X,Y)-only co-Z arithmetic. They also provide a relatively comprehensive comparative analysis with other methods, in which they evaluate their proposed method in Virtex-2 up to Virtex-6, without using any DSPs and BRAMs. In their most recent platform (i.e., Virtex-6), they consume 44.3k LUTs, operating at 221 MHz maximum frequency, and acquire 0.650 ms execution time.

Previously, Lai et al. [18] in 2012 also utilized a pipelined Montgomery multiplier and performed their ECPM using the addition-and-subtraction method. They also proposed three different types of operation scheduling, in which their fastest approach (namely with their Type-III scheduling) was then compared to other works for Virtex-2, Virtex-4, and Virtex-5 platform. The implementation on their latest platform utilizes 3657 slices, 10 DSPs, and 10 BRAMs, which yields 0.860 ms execution time and 263 MHz maximum frequency. Their result is largely surpassed by Ma et al., whose latency is nearly half of that of [18]. Additionally, Vliegen et al. [19] and Hu et al. focused on developing low-area implementation, in which [19] uses 1947 slices, 7 DSPs and 9 BRAMs (Virtex-II Pro) for achieving 68.17 MHz maximum frequency and 15.760 ms execution time while [20] only uses slices without any other components, topping at 9370 for a maximum frequency and latency of 20.44 MHz and 29.840 ms, respectively.

### 4.2. Result and Analysis of Unified ECC Architecture

Besides high-speed, our method also supports multi-curve domain parameters. For instance, different standards (e.g., P-256 from NIST [8], secp256k1 from SECG [37], SCA-256 from SM2 [38], and Brainpool256 from the German Brainpool standard [39]) would be able to be implemented with just a single ECC processor. Moreover, our processor does not incur any additional costs besides BRAMs when adding support for different curves.

Currently, our implementation supports up to 256-bit modulus size. Nevertheless, it can be easily extended to the larger modulus size since our proposed pipelined multiplier-accumulator, constructed based on Equation (14), is scalable due to the divide-and-conquer characteristics of the employed algorithm. Table 4 presents the comparison of our method to the other two proposals on unified architecture. As shown, it can be inferred that our approach is notably faster than other similar works of [40,41].

In [40], Amiet et al. focused on building a flexible ECC processor that accommodates arbitrary curves in short Weierstrass form. Their design mainly improves the Montgomery modular multiplier previously proposed by [42] to support the pipeline and utilizes a different mechanism for treating the carry result. They leverage DSP cores to parallelize point addition and point doubling operations. Realized on Virtex-7 FPGA, their fastest implementation, which uses a word size of 64, requires 6816 LUTs and 20 DSPs to yield in the maximum frequency of 225 MHz and runtime speed of 0.69, 1.49, 4.08, and 9.7 ms for 192, 256, 384, and 512-bit modulus, respectively.

Wu et al. [41] proposed a word-based modular division and utilized parallel point additions and doublings as well as pipelined scalable multiplications and modular reductions to achieve a fast and unified ECC implementation for five NIST primes. To support those primes, the authors employ a scalable multiplication algorithm to deal with integers of different lengths. Employing 8411 slices and 32 DSPs, this approach works in the frequency up to 310 MHz, achieving 0.296, 0.389, 0.526, 1.07, and 1.86 ms on NIST-192, 224, 256, 384, and 521-bit modulus size, respectively.

## 5. Conclusions

In this paper, we have proposed a high-speed and unified ECC processor that works for generic Weierstrass curves over GF(p) on FPGA. The speed is obtained by utilizing our fast pipelined Montgomery Modular multiplier (pMMM) for performing ECPM, constructed from our *n*-bit pipelined multiplier-accumulator, which combines schoolbook long and Karatsuba–Ofman multiplication, allowing the multiplication to be performed in parallel while maintaining a low complexity. Furthermore, digit multipliers are handled by DSPs, resulting in an even faster execution time. Additionally, we also propose to modify certain components to maximize the speed gain and the overall performance: employing our unified and pipelined Modular Adder/Subtractor (pMAS) for the underlying field arithmetic based on the work of [11], as well as implementing a more efficient yet compact scheduling of Montgomery ladder algorithm previously proposed in [22]. Moreover, the generic architecture employed by our pMMM module enables a unified ECC architecture that supports multi-curve parameters. The implementation in the 7-series FPGA: Virtex-7, Kintex-7, and XC7Z020, shows that our technique executes in 0.139, 0.138, and 0.206 ms, respectively, which is the fastest in literature for generic curves as far as we know. It is worth to mention that our current approach is extensible to support more curve parameters for up to 256-bit modulus size, by only incorporating additional BRAMs. Lastly, our method is also resistant to side-channel attacks, making it suitable for applications requiring high speed and SCA-resistant characteristics, such as for the use in autonomous vehicles.

## Figures and Tables

**Table 1:** Performance comparison of the proposed generic ECC processor for Weierstrass curve up to 256-bit modulus size.

Designs	Platform	Slices	DSP	BRAM	Max. Freq. (MHz)	Cycles	Time (ms)	Time x Area ${}^{a}$
	Virtex-7	6909	136	15	232.3		0.139	0.96
This work	Kintex-7	7115	136	15	234.1	32.3k	0.138	0.98
	XC7Z020	7077	136	15	156.8		0.206	1.46
Roy et al. [11]	XC7Z020	2223	40	9	208.3	95.5k	0.459	1.02
Bajard et al. [15]	Kintex-7	1630	46	16	281.5	172.3k	0.612	1.00
Asif et al. [14]	Virtex-7	18.8k (LUT)	-	-	86.6	63.2k	0.730	3.43 ${}^{b}$
Ma et al. [16]	Virtex-5	1725	37	-	291	110.6k	0.380	0.66
Lai et al. [18]	Virtex-5	3657	10	10	263	226.2k	0.860	3.15
Shah et al. [17]	Virtex-6	44.3k (LUT)	-	-	221	143.7k	0.650	7.20 ${}^{b}$
Vliegen et al. [19]	Virtex-II Pro	1947	7	9	68.17	1074.4k	15.760	30.68
Hu et al. [20]	Virtex-4	9370	-	-	20.44	609.9k	29.840	279.60

${}^{a}$ TA = Slices x Time; ${}^{b}$ TA = LUTs/4 x Time (Assume 1 slice has 4 LUTs).

**Table 2:** Performance analysis of proposed generic ECC processor (256-bit) on Kintex-7.

Operation	Clock Cycles	Latency @234.1 MHz (ns)
1 × Input Modular Addition	5	21.36
3 × Input Modular Addition	7	29.90
1 × Modular Multiplication	26	111.07
4 × Modular Multiplication	29	123.89
Modular Inverse	6911	29,523.79
Ladder Setup	131	559.63
One Step Ladder Update	97	414.38
Ladder Finish	7050	30,117.60
One ECC Scalar Multiplication	32,272	137,865.98

**Table 3:** Resource consumption of proposed generic ECC architecture on Virtex-7 field-programmable gate array (FPGA).

Resource	Used	Available	Utilization %
LUT	22,736	433,200	5.25
FF	12,511	866,400	1.44
Slice	6909	108,300	6.38
DSP48E1	136	3600	3.78
BRAM	15	1470	1.02

**Table 4:** Performance comparison of the proposed unified ECC processor for Weierstrass curve up to 256-bit modulus size on Virtex-7 FPGA.

Designs	Curve	Modulus Size (Bits)	Slices	DSP	BRAM	Max. Freq. (MHz)	Time (ms)
		192					0.119
This work	Any	224	7281	136	15 *	204.2	0.138
		256					0.158
		192					0.296
		224					0.389
Wu et al. [41]	NIST	256	8411	32		310	0.526
		384					1.070
		521					1.860
Amiet et al. [40]	Any	192	6816 (LUT)	20		225	0.690
		256					1.490
		384					4.080
		521					9.700

* Adding more curve parameters will only increase BRAM size without affecting number of Slices and DSPs.

## Data Availability

Not applicable.
